# Differential Signal-Amplitude-Modulated Multi-Beam Remote Optical Touch Based on Grating Antenna

**DOI:** 10.3390/s24165319

**Published:** 2024-08-16

**Authors:** Yanwen Huang, Weiqiang Lin, Peijin Wu, Yongxin Wang, Ziyuan Guo, Pengcheng Huang, Zhicheng Ye

**Affiliations:** 1 Department of Electronic Engineering, Shanghai Jiao Tong University, Shanghai 200240, China; ywhuang666@sjtu.edu.cn (Y.H.); guo.zy@sjtu.edu.cn (Z.G.); pchhuang@sjtu.edu.cn (P.H.); 2 Fujian Science & Technology Innovation Laboratory for Optoelectronic Information of China, Fuzhou 350108, China; linweiqiang@fjoel.cn (W.L.); 21014082017@stu.hqu.edu.cn (Y.W.); 3 Key Laboratory for Laser Plasmas (Ministry of Education), School of Physics and Astronomy, Shanghai Jiao Tong University, Shanghai 200240, China; wupeijin@sjtu.edu.cn; 4 Hangzhou Institute of Optics and Fine Mechanics, Hangzhou 311421, China

**Keywords:** optical interaction, remote multi-touch, grating antenna, differential amplitude modulation

## Abstract

As screen sizes are becoming larger and larger, exceeding human physical limitations for direct interaction via touching, remote control is inevitable. However, among the current solutions, inertial gyroscopes are susceptible to positional inaccuracies, and gesture recognition is limited by cameras’ focus depths and viewing angles. Provided that the issue of ghost points can be effectively addressed, grating antenna light-trapping technology is an ideal candidate for multipoint inputs. Therefore, we propose a differential amplitude modulation scheme for grating antenna-based multi-beam optical touch, which can recognize different incidence points. The amplitude of the incident beams was first coded with different pulse widths. Then, following the capture of incident beams by the grating antenna and their conversion into electrical currents by the aligned detector arrays, the incident points of the individual beams were recognized and differentiated. The scheme was successfully verified on an 18-inch screen, where two-point optical touch with a position accuracy error of under 3 mm and a response time of less than 7 ms under a modulation frequency of 10 kHz on both incident beams was achieved. This work demonstrates a practical method to achieve remote multi-point touch, which can make digital mice more accurately represent the users’ pointing directions by obeying the natural three-point one-line aiming rule instantaneously.

## 1. Introduction

In information displays, human–computer interaction plays a crucial role in the evolution of intelligentization [1]. From conventional mouse and keyboard setups to existing capacitive sensing touch and voice recognition input, human–computer interactions are becoming more and more efficient and convenient [2]. Currently, in small- and medium-sized screens, multi-point direct touch technologies, such as capacitive [3], resistive [4], surface acoustic [5], and infrared light-occluded [6], are satisfactory and widely used. These technologies can achieve accurate touch recognition via input from users’ fingers.

However, as screen sizes are enlarged to accommodate larger audiences, particularly in education and conference settings, the interaction range of larger screens has exceeded the human body’s physical capabilities. For this reason, existing infrared light occlusion and capacitive technologies relying on the direct contact of human fingers or pointers with the screens often proximally confine users to their devices and, moreover, some positions are not directly touchable. Therefore, remote control interactions that can extend the touch range of the human body are urgently needed.

Based on the working principles, the techniques of remote touch can be categorized into inertial sensing, imaging recognition, and optical touch. The typical devices based on inertial gyro technology are fly mice and cyborg rumble pads, in which the acceleration of the device’s movements is measured by the embedded gyros and then converted into the moving of cursors on the screen. By using gyros, users can remotely control display devices, such as with optical mice on desks, without physical limitations [7,8]. Inertial sensor-based interaction devices [9,10], which can facilitate the sensing of the user’s movements in free space, have advantages in visual reality and wearable devices. However, since locating cursors relies on the relative acceleration data of devices’ movements other than from the absolute indication points of the control devices, users must shake or swing the devices repeatedly until the cursor is moved to the desired positions on the screen, leading to an unconformable experience because of the delay in reaction and directional inaccuracy. It seems that inertial gyro-based interaction devices are more suitable for virtual reality than flat panel displays, where users do not need to see their hands in the real world.

As for imaging recognition technology based on cameras, the interaction between users and displays comes from the detection of the movements or gesture changes of the hands or fingers, which are usually affixed with markers to enhance the image recognition ability of the computers [11,12,13]. The drawback of computation vision-based interaction is that it requires the cameras to capture the movement of the directors in real time, and thus, the operation zones are limited by the camera’s field of view and focus depth, which necessitates confined motion.

Optical touch technology employs lights as input signals, where the position of the cursor is directly obtained via laser beams, obeying the people’s habit of three points–one line. In this case, the laser pointers act as an extension of the user’s hand, where the positions illuminated by the laser pointers are instantly coincident with the interaction points. Consequently, it is more intuitive, reducing the user’s learning threshold and enabling a more comfortable experience. For optical touch, there are two primary methods for achieving illumination positions according to the distribution of photodetectors. For the planar array type (PAT) [14,15,16], the optical sensing units are embedded into each pixel of the display panel. For the line array type (LAT) [17], the detectors are set around the four edges of transparent microstructured film. The microstructures on the film are used to trap the incident lights and convert them into four guiding modes propagating along the film in total internal reflection. Eventually, the four guiding beams reach the line detector arrays, where they are converted into electric signals. Finally, the illumination positions of the lights on the screen are reconstructed by the cross points between the two virtual lines obtained from the corresponding coordinates of the detector arrays. The LAT has the advantage of a lower cost than that of the PAT. However, the LAT suffers from a large amount of scattering loss, which hinders its applications in large displays. For the LAT’s issues, remote optical touch technology based on grating antenna light trapping by using laser beams as input signals has been proposed, and single-point touch has been demonstrated [18]. Although the abovementioned optical touch technology affords accurate and instant location of the cursors, ghost points are inevitable if more than one beam is input. Consequently, in the current remote optical touch systems, the realization of multi-point operation has not yet been achieved.

Since analog light inputs cause ghost points, we employed digital signals with distinct parameters on each incident beam to differentiate them. Specifically, in our scheme, pulse-width modulation (PWM) is applied on the beams, with each beam being assigned a unique pulse width. When these beams illuminate the phototransistors, they generate pulsed photocurrents corresponding to the specific modulation characteristics. The photocurrents, upon passing through reference resistors, are transduced into voltages, which are then further converted into digital signals by analog-to-digital converters. Consequently, since the digital voltage retains the pulse width of the original beam, the differentiation of those digital voltages allows computers to distinguish among the incident beams. In this process, remote multi-point optical touch is realized. This innovation not only integrates the advantages of multi-touch into free-space human–computer interaction but also creates a more natural and intuitive experience for the users.

## 2. Materials and Methods

**Figure 1:**
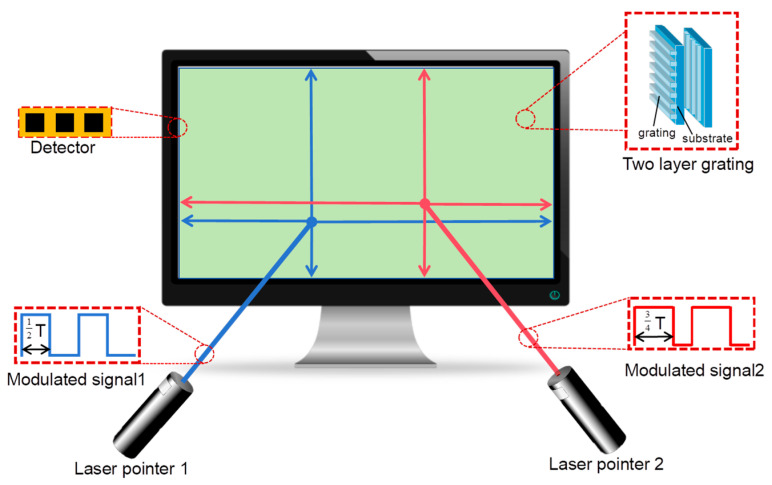
Schematic diagram of the multi-point optical interaction system. Pulse signals with different widths are modulated onto the infrared lasers.

The architecture of the multi-point optical interaction system is shown in Figure 1. The system consists of three principal components: two amplitude-modulated laser pointers for optical signal input, two layers of transparent optical waveguide film with grating antennas on the front surfaces, and optoelectronic detector arrays affixed to the perimeter of the waveguide films.

Different pulses are modulated onto the infrared laser pointers to divide inputs from multiple users, enabling mutual interference-free multi-user interaction. The dual layer of the optical waveguide films, in conjunction with optoelectronic detector arrays, are sandwiched between the backlight and the liquid crystal panel. One-dimensional grating antennas, with gating vectors oriented along the X direction on the first layer of film and the Y direction on the second layer of film, are configured to couple the incident lights into the waveguide and direct them to the sensor arrays. 

### 2.1. Design of Grating Antenna

The core component of this touch system is the subwavelength grating antennas [19,20], which are designed to couple the laser beams with a wavelength of 980 nm into the waveguide layers, thereby enabling the extraction of position information. The antennas satisfy the grating coupling equation shown in Equation (1), where n and n_i_ denote the refractive indices of the substrate and the air respectively, $\theta_{m}$ represents the diffraction angle of the lights propagating in the optical waveguide films, $\theta_{i}$ refers to the incidence angle in the air, m is an integer representing the order of diffraction, λ signifies the wavelength of the incident laser lights, and T denotes the period of the grating antennas.

(1) $$nsin\left(\theta_{m}\right)-n_{i}\sin\left(\theta_{i}\right)=\frac{m\lambda }{T}$$

**Figure 2:**
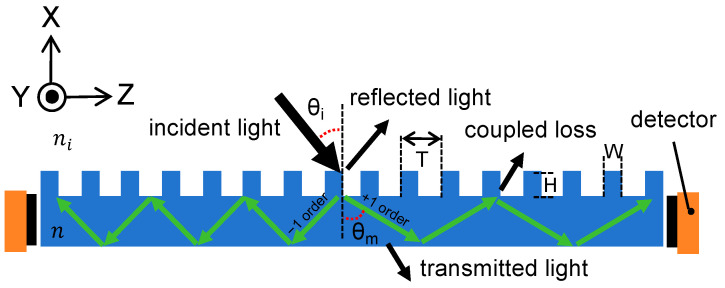
Diagram of light transmission in the grating antenna layer. The incident light from the free space is coupled with the grating antenna, and then converted into the confined light propagating along the waveguide. Finally, it is captured by the photodetectors positioned around the screens.

As shown in Figure 2, the incident laser light is coupled with the grating antenna to propagate along the optical waveguide films via total internal reflection (TIR). It is then detected by the photosensitive detectors located on the four edges of the film. It should be noted that the light propagating inside the waveguide will be partly lost outside of the waveguide at the TIR spots due to the grating diffraction. Thus, to minimize the re-coupling loss generated by the grating antenna, it is crucial to increase the $\theta_{m}$ according to Equation (1), which is discussed in detail in the following design section.

As depicted in Figure 2, the amount of light that reach the detectors around the edges of the optical films is decided by the coupling efficiency of the grating antenna and the coupling loss during transmission. The smaller the signal reaching the detectors, the more susceptible to noise interference the system becomes, and the more challenging it is to achieve an accurate resolution. Furthermore, there are periodical re-coupling losses at the TIR spots in the optical waveguide as mentioned above, so it is essential to decrease the re-coupling spots. According to Equation (1), when the light is obliquely incident to the grating surface, the diffraction angle of +1 order is larger than that of the −1 order, meaning that there is a longer step between two adjacent TIR spots, a smaller number of re-coupling spots, and thus, less loss. Consequently, only light with a +1 order diffraction was simulated and chosen to work as the waveguiding light signal.

**Figure 3:**
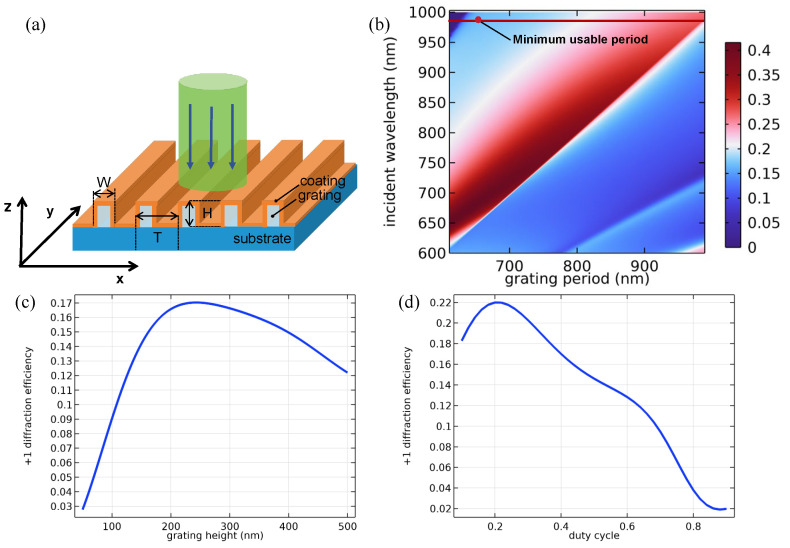
(**a**) Schematic diagram of the grating antenna and waveguide. (**b**) +1 order diffraction efficiencies of the grating antenna with incident wavelengths ranging from 350 nm to 1200 nm and grating periods of 600–1000 nm. (**c**) +1 order diffraction efficiencies of the grating with grating heights ranging from 0 nm to 500 nm and a grating period of 650 nm. (**d**) +1 order diffraction grating efficiencies with duty cycles ranging from 0.1 to 0.9 and a grating period of 650 nm.

Figure 3a illustrates the device structure of the periodical grating antenna, where each unit consists of an air cover layer, a coating layer (TiO_2_), a grating layer, and an optical waveguide film functioning as the substrate. The grating and the substrate are made of the same polycarbonate (PC) material. The numerical simulation and optimization of the grating antenna’s diffraction efficiency were conducted by using the finite element method based on COMSOL Multiphysics 6.16.0. In the simulation, the refractive indices of the air, TiO_2_ coating, and PC grating were 1.0, 2.3, and 1.57, respectively. Based on previous research [18], the duty factor and grating height H were initially set to 0.4 and 250 nm, respectively. The simulated +1 order diffraction efficiencies with different periods for normal incidence and wavelengths ranging from 350 nm to 1200 nm are illustrated in Figure 3b. As indicated by the red solid line with a dot in Figure 3b, in the pitch range of 630–980 nm under normal incidence, the grating antenna can convert the incident beams with a wavelength of 980 nm into a waveguide with +1-order diffraction. As mentioned above, the period of grating should be small enough to reduce the re-coupling loss; hence, a period of 650 nm was selected in our concept demonstration. Figure 3c,d illustrate the simulated +1 order diffraction efficiencies with the grating height changing from 50 nm to 500 nm, and the duty factor changing from 0.1 to 0.9. As presented in Figure 3c,d, at the highest diffraction efficiency, the grating height and duty factor were around 200 nm−300 nm and 0.1–0.3, respectively. However, owing to the limitation of the fabrication capability in this work, the exact grating height and duty cycle was hard to achieve; therefore, a grating height of 250 nm and a duty cycle of 0.25 were chosen.

### 2.2. Hardware Architecture

**Figure 4:**
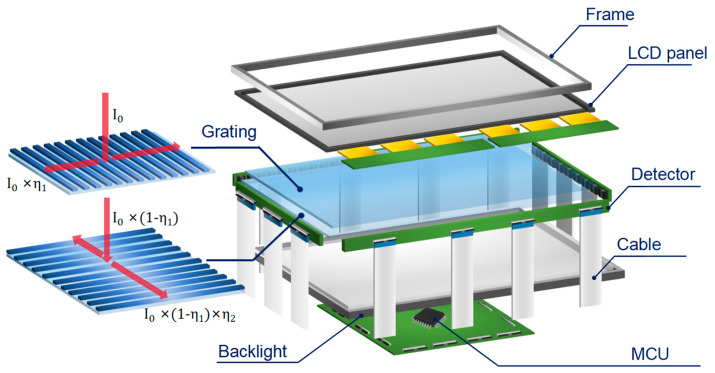
The diagram of hardware components and layout of the optical touch system. It includes an LCD panel, two layer of optical waveguide films, a backlight module, detector arrays, an electronic signal circuit, and a microcontroller unit.

The primary components of the prototype optical touch system are depicted in Figure 4. It consists of an 18-inch (365 mm × 235 mm, from HAEUVS, Shenzhen, China) liquid crystal display (LCD) panel, two layers of transparent PC optical waveguide films, a backlight module, a detection-sensing circuit, and a microcontroller unit (MCU). The grating antennas were fabricated by three main processes: photolithography, electroforming [21], and hot embossing [22]. Then, a layer of titanium dioxide was deposited onto the grating layer as a high-refractive-index coating to enhance the diffraction efficiency, thereby maximizing the optical signals reaching the photodetectors [23]. As illustrated by the red lines on the grating antenna, the incident infrared lights pass through the LCD panel at first and then illuminate the grating antenna, where they are coupled with the PC optical waveguide films via grating diffraction. The phototransistors (XL-3216PDC from Xinglight, Shenzhen, China) with a sensing area of 2.0 mm by 1.6 mm are used to catch the waveguide lights. They are organized into arrays, each consisting of 14 detectors spaced 3 mm apart. The quantum efficiency of the detector is around 80% at a wavelength of 980nm, as shown in Figure S1. To cover the entire display screen, around the optical films, seven arrays were placed at the top and bottom edges, and four arrays were placed on each of the left and right edges.

**Figure 5:**
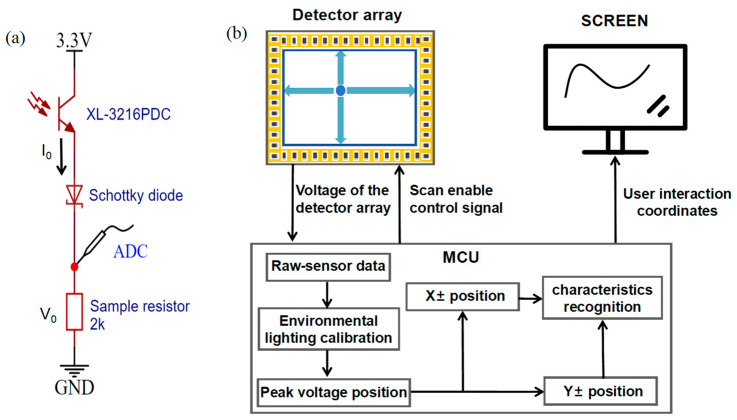
(**a**) The set-up of a detector unit consisting of a phototransistor, a Schottky diode, and a sampling resistor. (**b**) The working process of how the grating antenna-based position recognition module obtains the incident light positions and displays them on the screen, including the signal scanning of detector arrays, ambient light calibration, peak voltage position calculation, modulated signal feature identification, and interaction points display.

As depicted in Figure 5a, each detector unit consists of a phototransistor, a Schottky diode, and a sampling resistor. When the phototransistor is illuminated by the waveguide lights, a photocurrent $I_{0}$ is generated with period and duty cycles corresponding to the intensity of the light, producing a waveform that reflects the modulation pulses upon the incident light. Then, the photocurrent I_0_ consecutively passes through the phototransistor, the Schottky diode, and the sampling resistor. Owing to the negligible forward resistance of the Schottky diode, the photocurrent primarily generates a voltage V_0_ = I_0_*R_sam_ across the sampling resistor R_sam_. The voltage $V_{0}$ is subsequently converted into a digital voltage signal $V_{r}$ by the analog-to-digital converter (ADC), following the reception of an enabled signal.

Figure 5b demonstrates the entire interaction process via the grating antenna-based recognition module: First, the detector arrays are sequential activated by the MCU. Then, the digital voltage data are collected and processed to determine the positions of the interaction points, and finally, those points are displayed on the screen. During the operations, the ADC continuously samples the voltages of the sensors in each array four times to obtain a two-dimensional array of voltage signals $V_{r}(p,t)$, where p denotes the detector positions, ranging from 0 to 307, and t denotes the four consecutive sampling times, ranging from 0 to 3.

The raw signals are calibrated to eliminate the noise of ambient light. This calibration process mainly involves the subtraction of the detector voltage $V_{e}$ in the absence of laser illumination from the voltage data $V_{r}$. After calibration, the remaining voltage values $V_{s}$ exactly represent the laser signals. Then, among the voltage data $V_{s}$, the positions of the peaks are picked out. Subsequently, those peak voltage positions are grouped into two categories based on whether they align with the X axis or Y axis or not. The voltage characteristics of each peak are identified by continuous sampling results. The determination of the interaction points is achieved by drawing intersecting lines between the two peaks with identical voltage characteristics. Finally, the interaction points are displayed on the screen, indicating the users’ interested areas and aiding in the completion of the interactive process.

### 2.3. Modulation and Demodulation Methods

**Figure 6:**
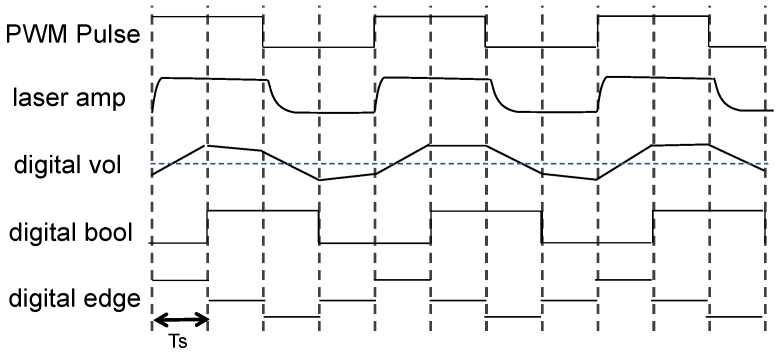
The diagram of how the signals are transmitted in the optical touch system. The periodic fluctuations in the laser amplitude, linked to the PWM signals, are transformed into digital voltage signals by sampling the detection circuit. Then, the digital voltage signals are converted into Boolean values through dynamic threshold judgments and classified by the rising and falling edges to demodulate the pulse’s width and frequency characteristics.

As illustrated in Figure 6 of the transfer process of the signals, first, the modulated pulses endow the amplitudes of the laser beams with distinct period and pulse width characteristics. Then, the characteristics of the laser amplitude are converted into digital voltage signals by the detection circuit. By demodulating and distinguishing based on the digital voltage characteristics, remote and simultaneous multi-user optical touch is realized.

To improve the response rate while maintaining the system’s capability of multi-user detection, we utilized duty cycle detection (DCD) to demodulate the pulse signals. As shown in Figure 6, dynamic thresholds, equivalent to half of the maximum voltage observed in the last four scans, are employed to convert the digital voltages into binary values. Those Boolean values are then categorized based on their transition trends: a change from 0 to 1 is identified as a rising edge (1), a transition from 1 to 0 is labeled as a falling edge (−1), and unchanged values represent a flat state (0). The intervals between rising and falling edges determine the demodulation results of the pulse width, whereas intervals between two consecutive rising edges constitute the modulation period.

**Figure 7:**
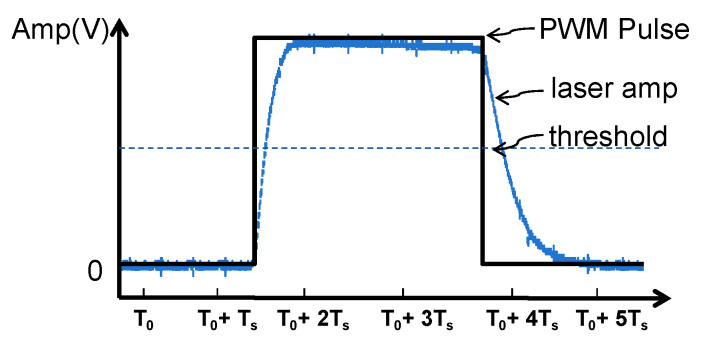
Distortion analysis of signal demodulation. The sum effect of the prolonged rising and falling times can result in pulse stretching or compression, leading to signal distortion.

During the demodulation process, the precision of the frequency and pulse width recovery depends on the fidelity of the digital voltage in replicating the characteristics of the pulse signal. However, the generation of the photocurrent necessitates a transient time to reach its peak and revert to the baseline dark current state, which are both 20 μs in this instance. The overlong rise and fall times result in a compression and stretching of the pulse respectively, thus constraining the selection of the modulation period and pulse width. Figure 7 exemplifies how the laser amplitude is distorted from the original waveform. The duration above the threshold constitutes the pulse width; thus, the signal distortion can be quantified by Equation (2).

(2) $$Signal\;\;Distortion=(T_{up}-T_{down})/(W\times T)$$

In Equation (2), T_up_ and T_down_ denote the rise and fall times of the detector, respectively. W represents the duty cycle of the modulation, while T indicates the modulation period. It is obvious that the discrepancy between the fall times and rise times generates distortion of the pulse pattern, leading to misidentification of the pulse width.

The establishment of the dynamic thresholds relies on the accurate capture of the peak voltages. As depicted in Figure 7, the duration of the peak is more likely influenced by the rise times. If the duration of the signal’s peak is less than the sampling interval $T_{s}$ of 25 μs, some of the peaks might be leaked, leading to the failed establishment of dynamic thresholds. To guarantee the acquisition of the peaks, the modulation signals must fulfill the following conditions:

(3) $$W\times T>T_{s}+T_{up}$$

In Equation (3), considering the sampling interval and rise edges, we opted for a pulse width of at least 45 μs to circumvent the sampling distortion. In addition, to surpass the minimum modulation pulse width difference of 25 μs, the pulse widths were set to 50 μs and 80 μs. Furthermore, to minimize the coding length and decrease the scanning times, the modulation period was set to 100 μs.

## 3. Experiment Process and Result Analysis

### 3.1. Experimental Setup

**Figure 8:**
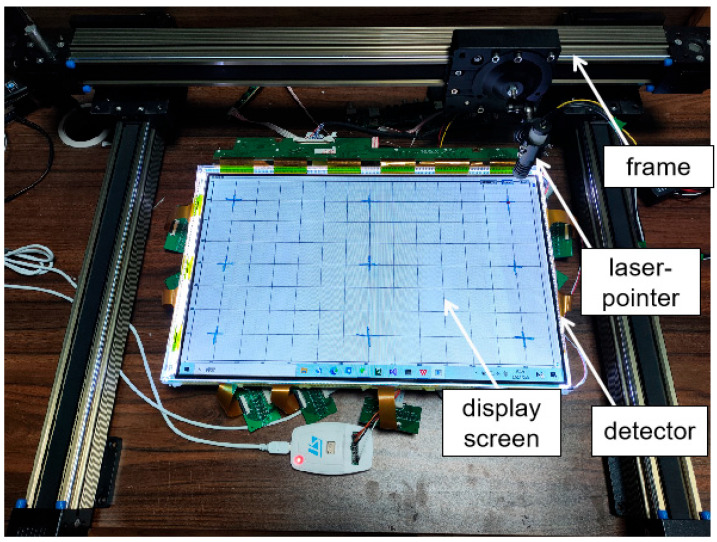
The experimental setup of the optical touch system, consisting of three movable rails, a laser pointer, a display screen, and a detector array. The laser pointer fixed on one of the Y-directional rails could scan the entire screen by moving the X or Y rails.

Figure 8 illustrates the measurement setup of the optical touch system, featuring the screen at its center, encircled by detector arrays. The arrays were directly interfaced with an MCU, which identified and displayed the interaction points on the screen. For the experiment, two infrared laser pointers emitting a wavelength of 980 nm, an operation voltage range of 2.0 V to 2.3 V, and a working current range of 600 mA to 800 mA were used. The amplitude modulation of the laser pointers was achieved through a PWM generator (HW-XHFSQ-1HZ-150KHZ from Shenzhen Infrared Laser Technology, Shenzhen, China). The generator was capable of producing pulse signals with adjustable duty cycles from 0% to 100% and frequencies ranging from 1 kHz to 100 kHz. A laser pointer was fixed on the Y-direction tracks, with its light beam perpendicular to the screen. During the experiment, the laser pointer could scan the entire screen by moving the tracks in the X and Y directions.

### 3.2. Static Accuracy Experiment

**Figure 9:**
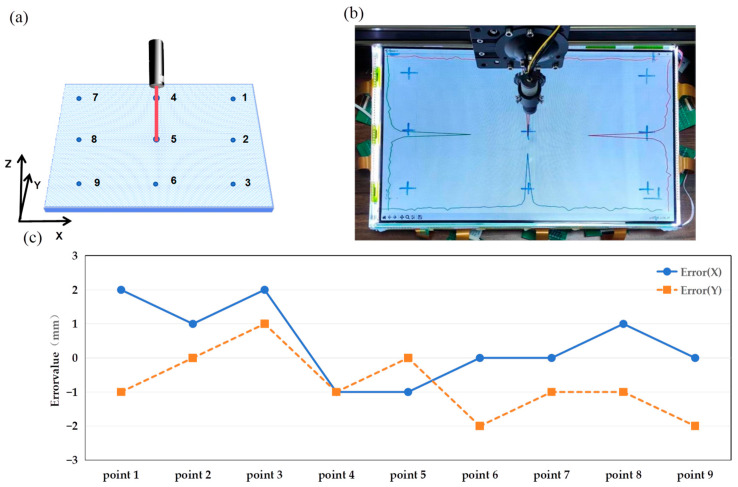
The diagram of the single-input experiments. (**a**) A 9-point measurement schematic to evaluate the position detection accuracy. (**b**) The response values of the sensor array when the laser pointer is illuminated to the position of No. 5. (**c**) Error evaluation of single input experiment.

As illustrated in Figure 9a, a measurement experiment involving nine equidistantly spaced points was employed to assess the detection errors across various screen positions. The target points were evenly distributed on the screen, with a spacing of 8 cm along the Y axis and 15 cm along the X axis. At each incident point, the calculated cursor positions were recorded for error analysis. The error metric is the distance between the intersection point and the actual incident point along the X and Y axes. In this proof-of-concept experiment, the incident light beams were perpendicular to the display plane.

Figure 9b presents one example of voltage sampling results, corresponding to the incident position labeled No.5. As expected, distinct peaks along the X and Y axes are evident. This demonstrates how the intersection point of two lines, drawn on the X axis and Y axis at the voltage peak positions, can serve as an interaction point.

The errors obtained from nine experiments are shown in Figure 9c. The maximum errors on both the X axis and the Y axis were 2 mm, with average errors of 1 mm. These errors are mainly attributed to the 3 mm distance between the detectors, even after applying the quadratic curve fitting method.

**Figure 10:**
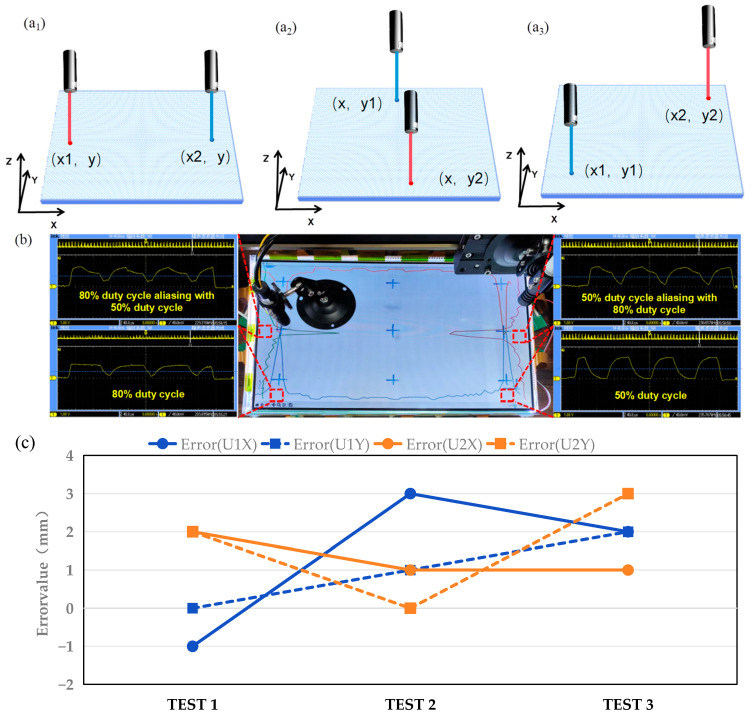
Two-point touch error measurements, including (**a1**) the same Y, (**a2**) the same X, and (**a3**) different X and Y coordinates. (**b**) The response values of the sensor arrays when the laser pointer is illuminated, as shown in (**a1**). (**c**) Error evaluation of dual input experiment.

To validate the effectiveness of multi-user recognition, pulses with a frequency of 10 kHz and different duty cycles of 80% and 50%, respectively, were modulated onto the two incident infrared laser pointers. As depicted in Figure 10a1–a3, three scenarios for two-point incidence were examined: instances with the same Y coordinate, those with the same X coordinate, and those with different X and Y coordinates.

A typical incidence corresponding to the incident positions of Figure 10(a1), is illustrated in Figure 10b. Two laser pointers are aligned on the same Y axis, leading to an overlapping signal area on the corresponding detector arrays. Nevertheless, the conventional cross-line intersection method, which is merely based on unmodulated zero-frequency signals, failed to distinguish between these inputs. Meanwhile, the waveform at the peak voltage positions, as depicted in Figure 10b, demonstrates that dynamic threshold judgment enabled the differentiation of the pulse width characteristics within the overlap area. Thus, duty cycle detection was used to ascertain characteristics, allowing intersecting lines to be drawn at the voltage peak positions with identical characteristics to determine the unique interaction positions. The voltage amplitude of the detector when two laser pointers were incident on the same point is shown in Figure S2. As shown in Figure 10c, the discrepancy between the displayed user coordinates and the actual positions was within 3 mm, ensuring satisfactory accuracy.

### 3.3. Interactive Performance Experiment

In the operation of optical touch systems, a user’s interaction trajectory is made up of discrete touch points, the spacing of which significantly affects the fluidity of the interaction. To determine the response speed limits of the optical touch system, interactive performance experiments were conducted under both single-point and two-point incidence conditions. In the single-point experiment, the screen was scanned by the laser along the X axis repeatedly at a speed of 0.5 m/s, and the computed touch points were recorded and displayed. In the two-point experiment, a second laser pointer with a different modulation characteristic was incorporated into the setup, while the rest of the processes remained unaltered. The scanning process was sustained for 20 s to facilitate a comprehensive assessment of the system’s continuity. The temporal intervals between the accurately identified points served as indicators of the response times, whereas erroneously identified points were annotated for calculating the recognition accuracy.

**Figure 11:**
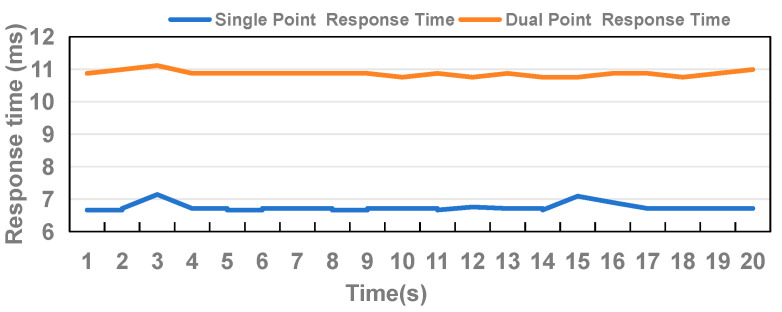
Comparative analysis of DCD method in single-point and dual-point scenarios with response time.

The results of the response speed in the single-point and dual-point experiments are shown in Figure 11. Under the single-point conditions, an average rate of 148 touches per second, corresponding to a response time of 6.75 ms per touch, was recorded. A total of 2963 touch points was recorded with only 17 misjudged points, resulting in a recognition accuracy of 99.4%.

Under the dual-point conditions, the display screen exhibited an average of 184 touch points per second for two users, i.e., 92 touch points per second for each user. This corresponds to a response time of approximately 10.8 ms per touch. During the entire process, a total of 3682 touch points were displayed, with only 17 misjudged points, resulting in a recognition accuracy of 99.5%. These results demonstrate that the DCD’s performance approaches the perceptible latency threshold of 6 ms [24], thereby meeting the requirements for user interaction.

**Figure 12:**
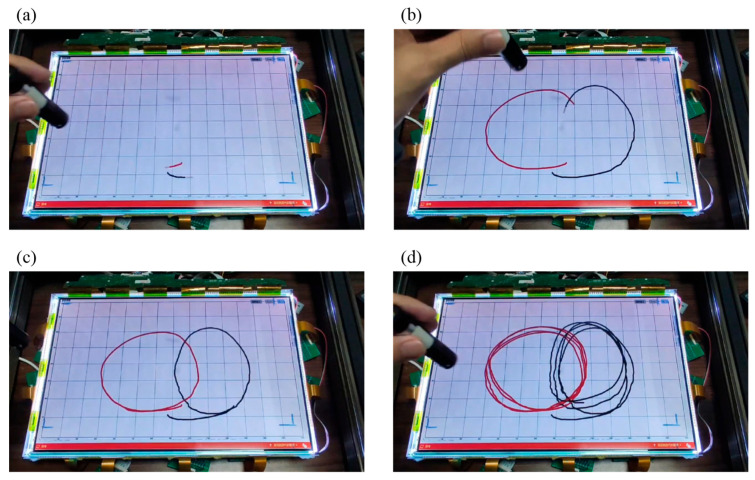
Demonstration of simultaneous dual-point writing. Two laser pointers were utilized to draw intersecting circles on the screen, with the display reflecting the recognition outcomes. The process is divided into four stages of (**a**) starting, (**b**) intersecting, (**c**) returning, and (**d**) repeating.

To demonstrate the inevitable crossover incidents in the multi-point input process, two laser pointers were utilized to draw intersecting circles on the screen. The writing demonstration with dual-point input is presented in Figure 12. The input process comprises four stages: initialization point recognition, intersection point identification, reversibility, and replicability. In the starting stages, as shown in Figure 12a, we began by drawing circles from the bottom of the screen, with the starting points of the two laser pointers identifiable as red and black. At the intersecting moment, as shown in Figure 12b, the circles drawn by the two laser pointers of different colors intersected, and the intersection points were correctly displayed in the respective color. In the returning state, as shown in Figure 12c, we completed the drawing of two circular trajectories, and both trajectories were displayed independently without confusion. To ensure reproducibility, we repeated the drawing of the intersecting circles with the two colors several times, as shown in Figure 12d. A smoother video demonstration can be viewed in Video S1. The experimental results indicate that DCD can identify crossover effects during operation, thereby confirming the feasibility of multi-input interaction.

## 4. Conclusions

We proposed a differential amplitude modulation scheme for grating-antenna-based remote multi-point touch, where the grating antennas act as signal transmission media. To facilitate simultaneous multi-user interaction, amplitude modulation upon the incident beam is employed to identify different users. To minimize distortion impacts and precisely demodulate the signals, the modulation period and pulse width were adjusted to match the electric character of the detectors during the signal conversion. The scheme was successfully validated on an 18-inch screen, where concurrent two-point optical touch identification was achieved with a location recognition error under 3 mm and a response time of less than 7 ms, yielding user-recognition accuracy exceeding 99.5%. In summary, our optical remote multi-touch technology opens a new pathway for future intelligent interaction, and we believe that by using photodetectors with shorter response times or faster sampling chips, the response time of the interaction can be much further improved, thereby enhancing the user experience even further.

## Data Availability

The raw data supporting the conclusions of this article will be made available by the authors upon request.

## Supplementary Materials

The following supporting information can be downloaded at: https://www.mdpi.com/article/10.3390/s24165319/s1, Figure S1. The quantum efficiency of the detector; Figure S2. The detector’s voltage amplitude when two laser pointers are incident on the same point; Video S1: Demonstration of simultaneous dual-point writing.
