# Supplementary material for: Differential Signal-Amplitude-Modulated Multi-Beam Remote Optical Touch Based on Grating Antenna
Source: Sensors (Basel). 2024 Aug 16;24(16):5319. doi: 10.3390/s24165319 (PMC11359848; doi:10.3390/s24165319)
Supplement: Supplementary file 1 [file sensors-24-05319-s001.zip › sensors-3052421-supplementary.pdf]

Supplement

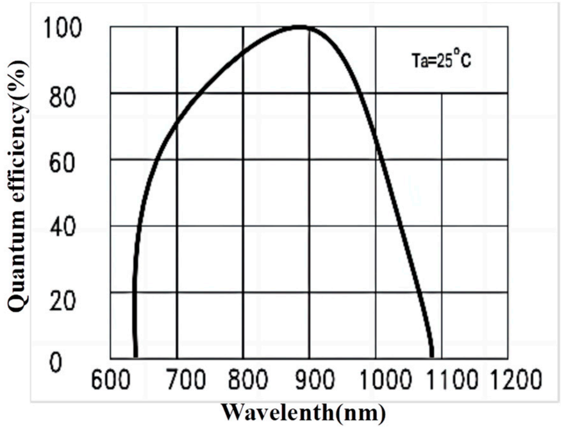

Figure S1. the quantum efficiency of the detector

Figure S1 illustrates the quantum efficiency of the detector. The x-axis represents the wavelenth of incident light, while the y-axis shows the quantum efficiency of the detector. As indicated by the graph, the detector exhibits a quantum efficiency of 80% at the 980nm wavelength, which is sufficient for our application.

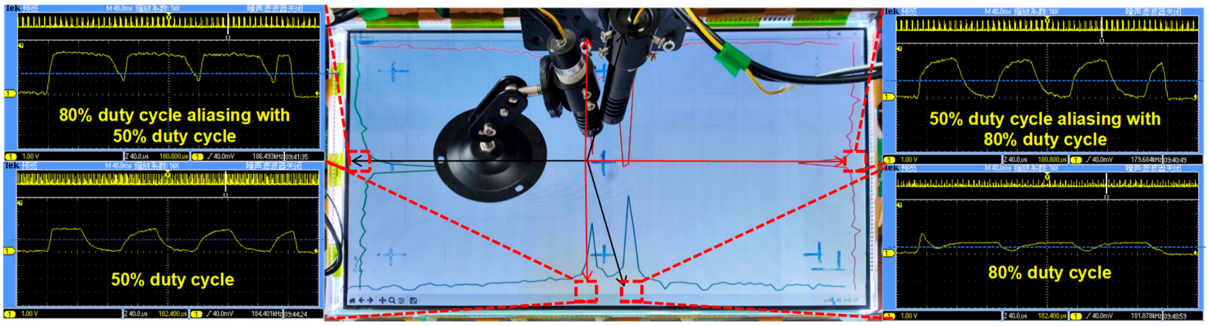

Figure S2. Detector's voltage amplitude when two laser pointers are incident on the same point

As depicted in Figure S2, two laser pointers are incident on the same point, with one on the left tilted with 80% duty cycle and the other on the right vertically incident with 50% duty cycle. The vertical laser pointer's light path is indicated by the red arrow, while the tilted laser pointer's light path is shown by the black arrows. The introduction of a tilt angle in the incident laser pointer leads to a deviation in the propagation direction, causing a shift in the interaction position. Additionally, due to the exceedance of the +1 order diffraction beyond the operational range with the tilt angle, most of the energy is concentrated in the -1 order, primarily on the left side.

From the figure, it is evident that the response points of the two laser pointers along the X-axis do not overlap, facilitating clear differentiation. On the Y-axis, since the -1 order diffraction concentrates most of the energy, dynamic thresholding allows us to distinguish the overlapping regions with different duty cycles on the left and right sides. This demonstrates the effectiveness of our system in distinguishing between the two laser pointers based on their respective light paths and diffraction patterns.
